# Opposing Roles of Interferon-Gamma on Cells of the Central Nervous System in Autoimmune Neuroinflammation

**DOI:** 10.3389/fimmu.2015.00539

**Published:** 2015-10-30

**Authors:** Payton A. Ottum, Gabriel Arellano, Lilian I. Reyes, Mirentxu Iruretagoyena, Rodrigo Naves

**Affiliations:** ^1^Immunology Program, Biomedical Sciences Institute, School of Medicine, Universidad de Chile, Santiago, Chile; ^2^Faculty of Science, Universidad San Sebastián, Santiago, Chile; ^3^Department of Clinical Immunology and Rheumatology, School of Medicine, Pontificia Universidad Católica de Chile, Santiago, Chile

**Keywords:** interferon-gamma, experimental autoimmune encephalomyelitis, multiple sclerosis, glial cells, neurons, central nervous system, neuroinflammation

## Abstract

Multiple sclerosis (MS) is the principal cause of autoimmune neuroinflammation in humans, and its animal model, experimental autoimmune encephalomyelitis (EAE), is widely used to gain insight about their immunopathological mechanisms for and the development of novel therapies for MS. Most studies on the role of interferon (IFN)-γ in the pathogenesis and progression of EAE have focused on peripheral immune cells, while its action on central nervous system (CNS)-resident cells has been less explored. In addition to the well-known proinflammatory and damaging effects of IFN-γ in the CNS, evidence has also endowed this cytokine both a protective and regulatory role in autoimmune neuroinflammation. Recent investigations performed in this research field have exposed the complex role of IFN-γ in the CNS uncovering unexpected mechanisms of action that underlie these opposing activities on different CNS-resident cell types. The mechanisms behind these two-faced effects of IFN-γ depend on dose, disease phase, and cell development stage. Here, we will review and discuss the dual role of IFN-γ on CNS-resident cells in EAE highlighting its protective functions and the mechanisms proposed.

## Introduction

Multiple sclerosis (MS) and its animal model, experimental autoimmune encephalomyelitis (EAE), are chronic autoimmune diseases of the central nervous system (CNS) characterized by inflammatory infiltrates, demyelination and neurological damage (1, 2). MS and EAE were initially considered to be mediated by interferon (IFN)-γ-expressing T helper (Th) 1 cells (3, 4). Currently, it is most widely accepted that several innate and adaptive immune cell types and immunomodulatory molecules contribute to the disease development and progression (5). Moreover, several studies have challenged the notion that IFN-γ is only pathogenic, and accumulative evidence attributes it a protective role in EAE and MS (6–8). In this same journal research topic, we have reviewed recent data supporting a stage-specific participation of IFN-γ in MS and EAE providing a plausible explanation for previous conflicting results and a model whereby this cytokine can both promote and limit the development of these pathologies (8). However, the majority of these studies have focused on the roles of IFN-γ in immune cells, while its activity in CNS-resident cells remains less explored. In this review, we will begin by discussing evidence that reports opposite roles of IFN-γ in the CNS during EAE development. Then, we will review both the inflammatory and protective effects of IFN-γ on glial cells and neurons in EAE.

## Opposing Effects of IFN-γ in the CNS during EAE

Early studies demonstrated that IFN-γ can activate CNS-resident cells and induce expression of major histocompatibility complex (MHC) molecules (3, 9). Direct injection of IFN-γ into the rat CNS induced inflammation and cellular infiltration similar to that observed in EAE (10–13) and potentiated the demyelination process (13). Furthermore, demyelination occurred in transgenic mice expressing IFN-γ under the control of the myelin basic protein (MBP) promoter (14–16). On the contrary, animals with EAE that were injected with IFN-γ systemically or directly into the CNS showed amelioration of clinical symptoms (17–20). Therefore, despite early data reporting an inflammatory effect of IFN-γ in the CNS during EAE development, accumulating evidence has also demonstrated a neuroprotective activity for IFN-γ in this disease.

Classical EAE is characterized by an ascending progressive paralysis dominated by inflammatory lesions in the white matter of the spinal cord and limited brain inflammation (2, 21). However, the absence of either IFN-γ or its receptor (IFNGR) leads to the development of atypical EAE symptoms such as head tilting, ataxia, dystonia, spasticity, and axial-rotation suggestive of brain-associated damage (19, 22–24). This atypical EAE is associated with greater encephalitogenicity of Th1 and Th17 cells and enhanced demyelination in the brainstem and cerebellum (19, 24). Even more, some studies have shown that IFN-γ may exert opposite effects in the brain and spinal cord determining the regional localization of lesions and inflammation in EAE. According to this evidence, IFN-γ induces inflammation in the spinal cord but protection in the brain (21, 25, 26). The mechanisms underlying these differential effects of IFN-γ may involve the regulation of the expression of specific chemokines in the brain versus spinal cord that restrain encephalitogenic T cell brain infiltration (19, 24, 25).

IFN-γ may also exert differential effects on the blood–brain barrier (BBB) and blood-spinal cord barrier (BSCB), resulting in opposite effects on their function and integrity. Supporting this hypothesis, atypical EAE symptoms were significantly ameliorated in mice that only expressed IFNGR in endothelial cells (EC-IFNGR mice). In these EC-IFNGR mice, inflammatory immune cell infiltration and demyelination were significantly inhibited in the brain but not in the spinal cord. In contrast to IFNGR-deficient mice, the functional integrity of the BBB was preserved in EC-IFNGR mice. This could be due to enhanced IFN-γ-induced claudin-5 expression that resulted in increased paracellular tightness of brain endothelial cell cultures (27). Therefore, these results indicate that endothelial expression of IFNGR is necessary for maintaining BBB function and preventing atypical EAE and brain inflammation. Further analysis must be performed in order to establish differential effects of IFN-γ on the BSCB.

## IFN-γ and CNS-Resident Cells

**Figure 1:**
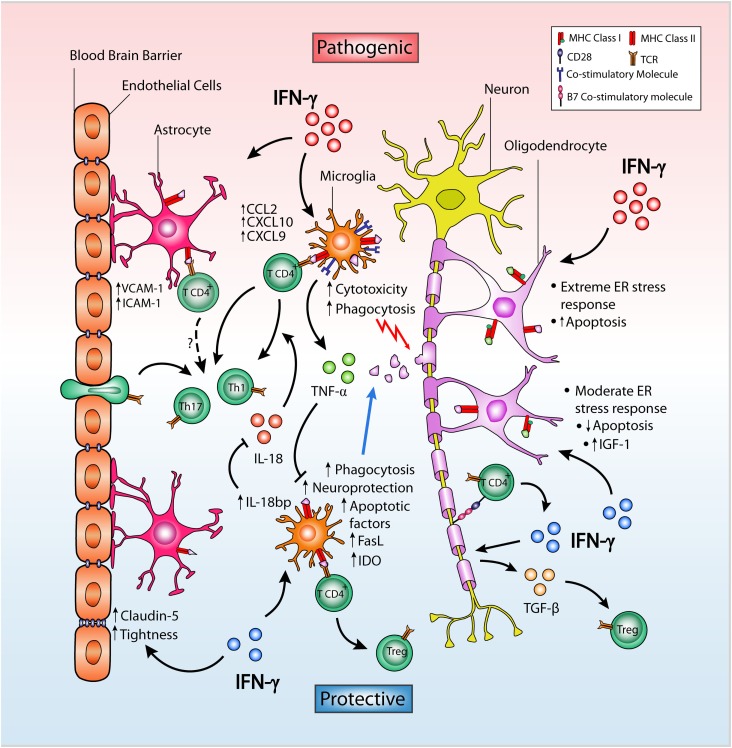
**Dual role of IFN-γ on CNS-resident cells in EAE**. IFN-γ in the CNS may facilitate helper T cell infiltration and neuroinflammation by inducing expression of VCAM-1, ICAM-1, and the chemokines CCL2, CXCL9, and CXCL10 in astrocytes located in close proximity to the BBB. Despite this, IFN-γ promotes BBB integrity by enhancing the expression and membrane distribution of claudin-5, in turn increasing tightness and ameliorating EAE severity. In microglia, IFN-γ differentially causes pathogenic cytotoxicity or neuroprotection and apoptosis depending on dose. Low-dose IFN-γ can induce tolerogenic microglia, while high doses result in increased expression of MHC class II protein and co-stimulatory molecules, leading to encephalitogenic T cell activation. TNF-α expression is upregulated by IFN-γ in microglia and inhibits their neuroprotective properties. In contrast, IFN-γ upregulates microglial IDO expression limiting T cell proliferation and inducing Fas and FasL expression favoring increased T cell apoptosis. IFN-γ also induces IL-18bp expression in microglia which inhibits the proinflammatory actions of IL-18 inhibiting EAE. In neurons, IFN-γ can induce B7 co-stimulatory molecules and TGF-β expression, promoting a Treg cell population that suppresses EAE. In oligodendrocytes, the effect of IFN-γ depends on both dose and the level of ER stress. By inducing expression of MHC molecules and other immune proteins, IFN-γ overloads the stress response in actively myelinating oligodendrocytes causing cell death, whereas a moderate stress response induced by low dose of IFN-γ on steady-state oligodendrocytes decreases apoptosis and increases IGF-1 levels in the CNS promoting remyelination of post-neuroinflammatory damage.

There are two groups of glial cells in the CNS: the macroglia, including astrocytes, oligodendrocytes, and ependymal cells, and the microglia. Several studies support the notion that glial cells not only provide functional support to neurons nor are they only a target of autoimmune injury, but are also active players in the development and progression of MS and EAE. Indeed, studies using an adoptive transfer EAE model have suggested that prevention of atypical EAE by IFN-γ is dependent on IFN-γ signaling not only in encephalitogenic T cells but also in glial cells (19, 25). Below, we will review the opposite effects of IFN-γ on glial cells and neurons, highlighting its less known protective functions and the mechanisms whereby IFN-γ is able to exert neuroprotection (Figure 1).

### Oligodendrocytes

Oligodendrocytes are CNS-resident myelin-producing cells and thus necessary for remyelination. Loss of oligodendrocytes, demyelination, and axonal damage are hallmarks of MS and EAE. Several *in vitro* studies have shown detrimental effects of IFN-γ on oligodendrocyte survival (28). Consistently, overexpression of IFN-γ in the CNS results in oligodendrocyte apoptosis and inhibition of myelination during development and after a demye-linating insult (15, 16, 29). However, when exposed to low levels of IFN-γ, oligodendrocytes were protected against oxidative stress and showed enhanced proteasome activity, two important processes preventing the accumulation of oxidized proteins, mitochondrial dysfunction and apoptosis (30). Furthermore, transgenic mice expressing low levels of IFN-γ were protected against chemically induced demyelination with cuprizone, and these animals did not show signs of oligodendroglial death, astrogliosis, or microgliosis compared to wild-type mice exposed to the toxin (31). Interestingly, elevated levels of insulin-like growth factor (IGF)-1 were detected in the CNS of these transgenic mice, which might contribute to the observed protective effects of IFN-γ, as IGF-1 has been demonstrated to inhibit oligodendrocyte apoptosis and promote myelination (31). Therefore, these results indicate that IFN-γ dose is critical in determining the survival of oligodendrocytes.

Those studies performed with transgenic mice have the limitation that overexpression of IFN-γ was induced during myelin formation whereas EAE and MS occur when myelination has been established in adulthood. In order to overcome this pitfall, the same authors developed an elegant *in vivo* experimental model that allowed temporally regulated delivery of IFN-γ to the CNS through a tetracycline-controllable system. IFN-γ delivery in the CNS that was first detected in the acute phase of EAE ameliorated disease severity and prevented oligodendrocyte loss, demyelination, and axonal damage. By contrast, delivery of this cytokine during the EAE remitting phase delayed disease recovery and inhibited remyelination (32). Interestingly, the effects of this cytokine in oligodendrocytes were mediated by the activation of protein kinase RNA-like endoplasmic reticulum kinase (PERK) and the phosphorylation of the α-subunit of eukaryotic translation initiation factor 2 (eIF2a), factors associated with the endoplasmic reticulum (ER) stress response (29, 32). The authors explained these apparently contradictory results proposing a dual role of IFN-γ on oligodendrocyte survival depending on their developmental stage and cellular stress (33, 34). According to this model, during the active process of myelination such as CNS development or remyelination (i.e., EAE remission), IFN-γ causes an overloaded ER stress response by increasing expression of MHC molecules and other inflammatory proteins in oligodendrocytes with already elevated ER stress, resulting in apoptotic program activation. Conversely, mature oligodendrocytes in adult mice produce significantly lower levels of membrane to maintain myelin homeostasis (i.e., acute EAE). In this case, IFN-γ would induce a moderate and protective ER stress response leading to oligodendrocyte survival and remyelination (33–35). Further investigations have determined that protection of oligodendrocytes in EAE may be mediated through Janus kinase (JAK) and the transcription factor signal transducer and activator of transcription (STAT)-1 signaling, as overexpression of the suppressor of cytokine signal (SOCS)-1, a competitive inhibitor of the IFN-γ-induced JAK/STAT-1 signaling pathway resulted in early EAE onset, enhanced inflammation, and oligodendrocyte apoptosis (30). Instead, the signal transduction pathway activating the transcription factor interferon regulatory factor (IRF)-1 would play a proinflammatory role in oligodendrocytes during EAE progression (36).

### Microglia

Microglia comprise 10–20% of all glial cells and are considered the immune sentinels of the CNS (37). They develop a broad and versatile range of functions involved in inflammation, immunomodulation, and promotion of neural repair that can be mediated in part by IFN-γ (38, 39). IFN-γ potentiates the phagocytic activity of microglia (40) and induces the expression of MHC class II and co-stimulatory molecules. This enables them to function as antigen-presenting cells (APCs) for infiltrating myelin-specific T cells leading to inflammation and demyelination (41–45).

During neuroinflammation, different subtypes of microglia can be distinguished according to their activation status. M1 microglia are primarily associated with an inflammatory phenotype and EAE initiation, while M2 microglia play an anti-inflammatory role and participate in tissue repair and remodeling associated with EAE recovery (5, 46). Despite being a potent activator of microglia and their polarization toward M1, IFN-γ can, at least in part, regulate the dual activity of microglia, modulating both pathogenic and regenerative processes (40, 47–49). How can IFN-γ lead these opposing roles?.

Evidence suggests that IFN-γ dose constitutes a fine-tune mechanism of regulation determining the balance between inflammatory and anti-inflammatory microglia. IFN-γ along with neuroantigen can determine an effector or regulatory helper T cell response modulating the activation state of microglia in a dose-dependent manner (48). Myelin oligodendrocyte glycoprotein (MOG)-specific CD4^+^ T cells co-cultured with microglia that were pre-activated with a high dose of IFN-γ and MOG peptide were primarily differentiated toward CD4^+^CD25^+^FoxP3^−^ effector T cells. Instead, microglia primed with a low dose of both IFN-γ and neuroantigen induced the expansion of stable CD4^+^CD25^+^FoxP3^+^ regulatory T cells (Tregs) capable of suppressing EAE after adoptive transfer (48). Furthermore, low concentrations of IFN-γ enabled microglia to perform neuroprotective functions such as clearance of glutamate, neuronal survival, neurogenesis, and, to a lesser extent, oligodendrogenesis in response to CNS insult (50–52). Remarkably, EAE disease onset was significantly delayed in mice that were stereotaxically injected with microglia activated *in vitro* by a low dose of IFN-γ into the cerebral ventricles 7 days after immunization (53). By contrast, high concentrations of IFN-γ rendered microglia cytotoxic and impaired their neuroprotective activities (50–53). Interestingly, the dose-dependent paradoxical effects of IFN-γ may be mediated by microglial production of tumor necrosis factor (TNF)-α. Neutralization of TNF-α, whose expression is upregulated in IFN-γ-activated microglia, boosted both the neurogenesis and oligodendrogenesis induced by microglia activated with low-dose IFN-γ, whereas the addition of TNF-α to similar cell cultures prevented these cell renewal processes (52). Thus, low levels of IFN-γ induce beneficial effects on microglia, which are counteracted by the microglial production of TNF-α in response to increasing levels of IFN-γ.

On the other hand, IFN-γ can induce several self-limiting negative feedback mechanisms to restrain the magnitude and duration of its proinflammatory effects on microglia and to provide CNS protection. For example, IFN-γ induces mRNA expression of SOCS-1 which antagonizes IFN-γ-induced STAT-1 activation (54). Indeed, overexpression of SOCS-1 inhibited IFN-γ-induced expression of MHC class II and CD40 in microglia by inhibiting STAT-1-mediated expression of the class II transactivator (CIITA) transcription factor (54, 55). Furthermore, IFN-γ induced microglial cell death through the upregulation of apoptotic proteins, especially bcl-2-associated X protein (Bax), in EAE (56). This process was only observed in the advanced stage of EAE, suggesting a disease stage-specific role of IFN-γ on microglia in EAE, and likely MS. In fact, the activation and subsequent death of microglia induced by IFN-γ was proposed as a possible mechanism underlying MS relapse and remission (56).

Another important regulatory mechanism of IFN-γ is the suppression of T cell functions by localized catabolism of the aminoacid tryptophan, which is essential for cell growth and functioning. Indoleamine-2,3-dioxygenase (IDO), whose expression in microglia is upregulated by the IFN-γ-induced STAT-1 and phosphatidylinositol 3-kinase (PI3K) signaling pathways (57), catabolizes tryptophan to kynurenine, and has been shown to play a protective role in EAE (58). Microglial IDO expression, induced by IFN-γ, reduced extracellular tryptophan and increased kynurenine which suppressed the proliferation of myelin-specific T cells and inhibited production of proinflammatory Th1 cytokines (59). In addition, tryptophan deprivation made T cells more susceptible to apoptosis through Fas-Fas ligand (FasL)-mediated signaling (60), whose expression can be induced in microglia by IFN-γ (61), enabling them with an additional protective mechanism by facilitating apoptosis of myelin-specific T cells.

Additionally, IFN-γ establishes a regulatory feedback limiting the inflammatory activity of interleukin (IL)-18, a member of the IL-1 family produced by microglia that synergizes with IL-12 to induce Th1 polarization and IFN-γ production. IL-18 is upregulated during EAE (62) and its neutralization inhibited EAE (63). In turn, IFN-γ induced the expression of IL-18 binding protein (IL-18bp), an endogenous inhibitor of IL-18, in CNS-resident microglia and infiltrating macrophages during EAE (64). Interestingly, augmented IL-18bp expression in the CNS mediated by an adenoviral vector, blocked the induction of Th17 cells, but not Th1 cells, in the CNS and significantly reduced the incidence and severity of EAE (64). Therefore, these results suggest that IFN-γ-dependent IL-18bp production in microglia might regulate the balance between the Th1 and Th17 responses during autoimmune neuroinflammation.

### Astrocytes

Astrocytes are the most abundant glial cell population and are essential for brain homeostasis and neuronal function. They play a major role in maintaining both the structure and functional integrity of the BBB, develop metabolic functions, and seclude damaged areas in the CNS. Astrocytes also exhibit a variety of immune functions whereby they may both stimulate and restrain neuroinflammation (65–68). Although the capability of astrocytes to function as APCs *in vivo* is still controversial, some investigators have identified astrocytes expressing MHC class II molecules (69), co-stimulatory B7 molecules (70), and intercellular adhesion molecule (ICAM)-1 (71) at the edges of active MS lesions. The expression of these antigen-presenting molecules in astrocytes is upregulated by IFN-γ both *in vivo* and *in vitro* (12). In line with these results, astrocytes exposed to IFN-γ *in vitro* can induce proliferation of myelin-specific T cells and Th1 differentiation (66, 68). Importantly, CD4^+^ T cells activated by astrocytes treated with IFN-γ and pulsed with myelin protein were able to induce EAE by adoptive transfer (72). In addition, IFN-γ upregulated expression of ICAM-1 and vascular cell adhesion molecule (VCAM)-1 on primary astrocytes (72, 73), which might have a direct impact on the extravasation of T cells into the CNS considering the close apposition of astrocyte foot processes to the microvascular endothelium of the BBB.

IFN-γ signaling in astrocytes also plays an important role in triggering expression of a wide range of chemokines involved in the recruitment of inflammatory cells to the CNS in EAE and MS. At the edges of demyelinating MS lesions, astrocytes expressed CCL2, CXCL10, and their respective receptors CCR2 and CXCR3 (74, 75). In EAE, CCL2 and CXCL10 were localized predominantly in astrocytes surrounding inflammatory lesions (76–78) and their expression was abolished in IFNGR-deficient mice (76). *In vitro* studies have confirmed that both human and murine astrocytes are induced by IFN-γ to produce CCL2, CXCL9, and CXCL10 (79–81).

Selective silencing of IFN-γ signaling in astrocytes using a lentiviral vector significantly ameliorated both actively and passively induced EAE (82). Surprisingly, purified astrocytes from spinal cord of these animals not only exhibited reduced expression of IFN-γ-inducible chemokines such as CXCL9, CXCL10, and CXCL11 but also inhibited expression of chemokines induced by IL-17 signaling such as CXCL1, CXCL2, and CCL20 (82). These results were confirmed *in vitro* by analyzing astrocytes deficient in IFNGR and suggest that IFN-γ signaling is the common pathway in both Th1 and Th17 cell-mediated EAE (82). In contrast to results depicted in astrocytes, the authors of this study found that selective blocking of IFN-γ signaling in microglia resulted in more severe EAE progression that was associated with significantly enhanced CNS inflammatory infiltration. In addition, IFN-γ signaling restricted microglia proliferation (82). Together, these results suggest that IFN-γ would exert opposing roles on two different CNS-resident cell types in EAE: inflammatory on astrocytes and protective on microglia.

However, another study using transgenic mice expressing a signaling deficient dominant-negative IFNGR1 specifically on astrocytes reached opposite conclusions when analyzing EAE progression (83). Compared to wild-type mice, these animals did not show alteration in the incidence, disease onset, or initiation of clinical symptoms. Instead, during the transition from acute to chronic disease, the clinical score and mortality were significantly increased, suggesting that IFN-γ signaling in astrocytes provides disease stage-specific protection (83). Different methodological strategies used to ablate IFN-γ signaling might explain the discrepant results found between these two studies (82, 83). A posterior study demonstrated that persistent CNS inflammation and progressive disability observed in these mice lacking IFN-γ signaling in astrocytes was associated with elevated expression of IL-6 and sustained proliferation and activation of microglia (84). Taken together, these results indicate that IFN-γ signaling plays an important role in controlling the proliferation of microglia during EAE either directly on microglia (82) or indirectly through astrocytes, (84) and highlight the crucial role of IFN-γ in the signaling network between astrocytes and microglia during EAE pathogenesis.

### Neurons

There is discrepant evidence regarding the relationship between neurons and IFN-γ, mainly described in the non-inflammatory brain, with both protective and detrimental effects (3, 9, 85, 86). In EAE, it has been observed that treatment with nerve growth factor (NGF), a well-known neural factor involved in neuron survival, decreased disease severity, and increased IFNGR expression in spinal cord neurons (87). Even more, neurons were induced to express transforming growth factor (TGF)-β and B7 molecules in response to IFN-γ produced by encephalitogenic T cells. Remarkably, encephalitogenic T cell–neuron interaction induced higher neuron survival and promoted the conversion of encephalitogenic CD4^+^ T cells to functional CD4^+^CD25^+^TGF-β^+^CTLA-4^+^ Tregs. The adoptive transfer of these converted Tregs suppressed EAE. Strikingly, the acquisition of this regulatory phenotype occurred in the CNS and was mediated by the production of IFN-γ and TNF-α by T cells (88).

## Concluding Remarks

Despite several studies having reported an inflammatory effect of IFN-γ in the CNS during EAE development, accumulating data have also demonstrated that IFN-γ possesses neuroprotective activity in this disease. The recent evidence summarized in this review underscores the duality of IFN-γ on CNS-resident cells in EAE and provides different mechanisms whereby this cytokine exerts these opposing activities. In low doses, IFN-γ induces protection in both microglia and oligodendrocytes, while in high doses it induces disease worsening effects in both glial cell types. In astrocytes, the evidence reveals a primarily disease-promoting role for IFN-γ signaling, although it may be disease stage-specific. Interestingly, IFN-γ also exerts opposing roles in oligodendrocytes depending on the cell maturation status and cellular stress response. Importantly, these findings implicate that defects or fluctuations related to the expression of IFN-γ, its receptor, and/or its signaling pathway may underlie the immunopathogenesis of MS as well as other demyelinating inflammatory diseases. Therefore, delineating the precise role of IFN-γ in the CNS and on CNS-resident cells might provide the basis for fine-tuning the development of CNS-targeted selective therapy.

## Conflict of Interest Statement

The authors declare that the research was conducted in the absence of any commercial or financial relationships that could be construed as a potential conflict of interest.
